# Effects of Silicon Compounds on Biomineralization, Osteogenesis, and Hard Tissue Formation

**DOI:** 10.3390/pharmaceutics11030117

**Published:** 2019-03-12

**Authors:** Werner Götz, Edda Tobiasch, Steffen Witzleben, Margit Schulze

**Affiliations:** 1 Department of Orthodontics, Oral Biology Laboratory, School of Dentistry, Rheinische Wilhelms University of Bonn, Welschnonnenstr. 17, D-53111 Bonn, Germany; wgoetz@uni-bonn.de; 2 Department of Natural Sciences, Bonn-Rhine-Sieg University of Applied Sciences, D-53359 Rheinbach, Germany; edda.tobiasch@h-brs.de (E.T.); steffen.witzleben@h-brs.de (S.W.)

**Keywords:** alveolar bone, biomineralization, osteoblast, osteogenesis, scaffold, silicon, silicates, stem cells, tissue engineering

## Abstract

Bioinspired stem cell-based hard tissue engineering includes numerous aspects: The synthesis and fabrication of appropriate scaffold materials, their analytical characterization, and guided osteogenesis using the sustained release of osteoinducing and/or osteoconducting drugs for mesenchymal stem cell differentiation, growth, and proliferation. Here, the effect of silicon- and silicate-containing materials on osteogenesis at the molecular level has been a particular focus within the last decade. This review summarizes recently published scientific results, including material developments and analysis, with a special focus on silicon hybrid bone composites. First, the sources, bioavailability, and functions of silicon on various tissues are discussed. The second focus is on the effects of calcium-silicate biomineralization and corresponding analytical methods in investigating osteogenesis and bone formation. Finally, recent developments in the manufacturing of Si-containing scaffolds are discussed, including in vitro and in vivo studies, as well as recently filed patents that focus on the influence of silicon on hard tissue formation.

## 1. Introduction

Chemically, silicon (Si) is a tetravalent metalloid found in group 14 in the periodic table, in the same neighborhood as carbon, but possessing comparably low reactivity. Apart from oxygen, Si is the most abundant element in the earth’s crust [1,2]. By mass, silicon is the eighth most common element in the universe, mostly occurring in its oxidized form as silica/silicate minerals and very rarely occurring as a pure element. Due to its high chemical affinity for oxygen, silicon forms various oxides and silicates. Thus, Si and corresponding compounds are essential natural nutrients responsible for many biological processes, including the regulation of bone metabolism [3,4]. Within the last decade, several research groups have studied the influence of Si and silicates on bone regeneration, focusing on different aspects (i.e., Si deficiency and dysfunctional bone metabolism [5,6,7], ion-doping [8], and protein template-mediated bottom-up approaches to promote bone formation [9,10,11,12]).

In this review, we first discuss aspects such as the bioavailability, metabolism, and toxicity of Si compounds. Then, mineralization mechanisms are presented involving silicon and silicates, followed by analytical techniques, in particular X-ray scattering methods used for natural and artificial bone characterization. Third, conventional and nanobased approaches to Si-containing scaffold manufacturing are discussed, including recently published data, filed patents, and commercial products for hard tissue formation.

## 2. Metabolism and Toxicity

### 2.1. Sources and Bioavailability

SiO_2_ is found in nature as amorphous biosilica in algae, sponges, and plants, providing a basis for intricate exoskeletons (“phytolithic”) [13,14]. Soluble silica acid ([Si(OH)_4_]) is the monomeric form of silica (orthosilicate), while the hydrated form, SiO_2_(H_2_O)X, can be found in water and also in plants and shows good water solubility and availability [15]. Hydrated Si can bind to organic molecules or form complexes with other inorganic compounds. There are different foods and beverages with a high Si concentration. Among them are cereals, rice, dried fruits, beer, beans, and spinach. In addition, some mineral and healing waters have a high concentration. Beer represents one of the beverages with the highest silica content due to high Si concentrations in hops and barley [16]. Amorphous Si formed in living systems is also called biosilica and provides the inorganic scaffold of skeletal elements in sponges (Desmospongiae) and diatomees [17,18]. The synthesis of biosilica is triggered by an enzyme, silicatein, which catalyzes Si polycondensation [18,19,20].

#### 2.1.1. Uptake

There are different sources from which soluble silicates can be taken up, e.g., drinking water, eating plants or beverages or meat, and also from soil or dust [1,14,21]. In Europe and North America, the mean uptake of Si ranges between 12 and 62 mg/day, while the mean Si uptake in East Asia is higher due to the higher amount of plants in nutrition [22]. Uptake from low molecular silicates is the best. Therefore, the Si availability from cereals, beer, and mineral water is the highest [1,23]. It is thought that nutritional amounts of Si can prevent abnormalities in bone formation and metabolism and collagen metabolism, with beneficial effects of Si resulting from a daily intake of 10–25 mg [24].

#### 2.1.2. Metabolism

Orthosilicic acid Si(OH)_4_ is the main Si species in man. After uptake, it is gastrointestinally absorbed and transported in the blood, mainly unbound. Only small amounts form complexes with Fe or Al at a neutral pH [1]. In the human body, Si amounts to 1–2 g, which corresponds to 0.01% of body weight, i.e., lower than Fe and Zn [1,25]). The concentration in serum ranges between 24 and 31 μg/dL. Outside the blood compartment, it is mainly bound to gycosaminoglycans. It has been speculated that serum and tissue levels of Si might be regulated by responsive elements or transporters, since such transporters (SITs) have been identified in plants and silicified organisms, (e.g., in diatoms or sponges [26,27]). Recently, an SIT named SLc34a2 was found in mammals [28]. Water channel aquaporins are homologous to SITs in rice, and have been detected in the small intestine and renal epithelia, and also in the bones and joints of mice. Their expression seemed to be diet dependent: Under a Si-rich diet, certain aquaporins were upregulated in kidney and calvarial bones [29]. However, how silicic acids reach their final site of deposition in the body still has to be investigated.

#### 2.1.3. Excretion

Si is excreted mainly renally after glomerular filtration and can be detected in the urine. It is possible that Si levels in urine could represent a parameter for Si bone metabolism, since reduced excretion could be associated with osteopenia [1]. After uptake, most absorbed Si is excreted after 4–8 h in urine [24]. In healthy human volunteers, the ingestion of soluble Si results in the excretion of the same quantity of Si within 24 h [30].

### 2.2. Functions

Detailed insights into the biological roles of Si are still lacking, although Si can be found in nearly all organs and tissues, with the highest concentrations in connective and hard tissues, including bone [1,14,15]. It has also been discussed that Si may exert influence over the metabolism of different organs or during inflammatory processes (see below) indirectly by altering the absorption and utilization of other trace elements [24]. The health benefits of Si for humans, especially under stress conditions, are well known. However, its probable role as an essential mineral is still under discussion [21].

#### 2.2.1. Connective Tissue

Si can be found in high levels in the extracellular matrix bound to different components, especially glycosaminoglycans [24]. The role of Si in connective tissue development and differentiation has been discussed, since Si can form complexes with polyols like hexosamines, which are components of gylcosaminoglycans and mucopolysaccharides that form extracellular matrix components. Additionally, Si plays a role as a cross-linking element in the bridging between proteoglycans and collagens [2,14,15,31]. Si supplementation in the diet shows stimulatory effects on cartilage synthesis [2]. In the connective tissues of rats, Si concentration decreases with increasing age. Probably, Si is necessary in young animals for connective tissue and bone development [32].

#### 2.2.2. Bone

Due to many in vitro and in vivo studies, it can stated that Si is benficial for bone tissue strcuture and function and is associated with calcium in bone metabolism [1,14,19]. There is increasing evidence that Si has a positive impact on bone homeostasis [33]. In older studies, Si deprivation led to abnormal growth and growth defects, e.g., in chickens [1,2,14]. More recently, this was based on different animal studies where Si application increased bone density and bone turnover in osteopenic ovariectomized rats, especially when the animals were fed with Ca-reduced food [24]. In a recent study by Jugdaohsingh et al. [34], a positive correlation between Si concentration in serum, bone quality, and osteocalcin levels in the serum of female rats was found. For humans, different clinical studies have shown correlations between bone health and Si levels. The Framingham Offspring Cohort has investigated the correlation between Si supply from food and hip and lumbar vertebral bone mineral density (BMD) measured by osteodensitometry in 1251 men and 1596 women. Positive correlations were found for men and premenopausal women [35,36]. During the Aberdeen Prospective Osteoporosis Screening Study, women ages 45 to 54 were observed for bone density and biochemical markers of bone metabolism, such as the anabolic procollagen Type 1 N-terminal propeptide (PINP) and catabolic PYD/DPD measured in serum and urine, respectively, and these factors were correlated with Si intake. In the group with the lowest Si intake, mean bone hip density was significantly lower than in the group with the highest intake. Si intake was negatively correlated with PY/DPD, indicating bone resorption, and was positively correlated with PINP, indicating bone apposition. However, these associations were not found in women with postmenopausal estrogen deficiency [37]. Spector et al. finally investigated the effects of silica substitution in different concentrations given in addition to calcium–vitamin D supplementation in 134 women with osteopenia for 12 months, and measured the N-terminal propeptide of procollagen type I as an indicator for anabolic effects [38]. Collagen synthesis was higher in women taking 6 and 12 mg of silica after 6 and 12 months, although no differences could be obtained for bone density measurements. These studies show that there is a correlation between the effects of Si on bone metabolisms and estrogen as well as on collagen metabolism. Taken together, it can be proposed that Si levels are associated with BMD, bone mechanics, and probably estrogen status [1,2,14]. In rat bones, the highest Si concentrations have been found in the low-mineralized bone of younger animals. An equal distribution in the mineral and collagen fractions of bone was shown, especially in the early stages of mineralization, which is also indicative of a close association between collagen and Si. With increasing age, total bone Si content increased [39]. Physiologically, hydroxyapatite can be substituted by other ions, including Si. Biological apatites can contain small levels of impurities, including Si [14,40].

Si can promote bone formation. In MG63 osteoblasts, Si stimulation has led to cell proliferation and remarkebly enhanced gene expression of collagen type I, the effects of which were theorized to be induced by induction of the extracellular signal–regulated kinases (ERK) pathway [41]. A conditioned medium supplemented with orthosilicic acid increased the secretion of collagen type I, alkaline phosphatase, and osteocalcin [42]. Increased expressions of different bone and osteogenesis genes in mice and men, such as bone morphogenetic protein-2 (BMP-2), collagen type I, and runx-2, have also been found [43,44,45,46]. Si is involved in the early stages of biomineralization, with high levels present during early calcification processes [1,14]. Probably, Si(OH)_4_ is able to induce the precipitation of hydroxyapatite (HA) from electrolyte solutions [2]. In vitro, Si-based components can cause an alteration of the expression of genes for amelogenin, ameloblastin, and enamelin in human osteoblats-like SaOS-2 cells, which are structural components of tooth enamelum [47].

Si inhibits the activity of macrophages and osteoclasts [14] and stimulates osteoprotegerin (OPG) in osteoblast-like cells, counteracting the catabolic effects of receptor activator of nuclear factor κB ligand (RANKL), which is involved in the activation of osteoclasts [48]. In the context of cross-talk between osteoblasts and osteoclasts, Si is thought to be inhibitory for the differentiation and activation of osteoclasts [49,50].

#### 2.2.3. Skin

Since orthosilicic acid has stimulated the synthesis of collagen type I and differentiation in human osteoblast-like cells in vitro [41,42], Si influencing collagen metabolism in the skin may be a reality. Indeed, oral intake of stabilized silica increased the elasticity and aesthetic appearance of facial skin in older women with UV-damaged skin in a randomized double-blinded clinical study [51]. There was also an increase in the brittleness of hair and nails. Positive effects on nails and hair were also found in another study with 24 women taking 9 mg of silicate per day versus a placebo for 9 months [52].

#### 2.2.4. Vessels

In an older study, a lower Si content was detected in the wall of the aorta of older people with structural changes, reduced elasticity, and increased stiffness [15]. Animal studies have shown that Si is a candidate for protecting atherosclerosis, especially related to age. However, anti-atherosclerotic effects seem to be species-related, since they could be observed in the fat-feed rabbit, but not in the mouse [39]. Applications of Si-rich drinking water in mice have induced the activity of endothelial NO synthetase in the aorta, which is involved in, e.g., the relaxation of smooth muscle cells in the vessel wall [53]. Findings from studies in silica-based bioactive glasses and calcium silicate ceramics have indicated that silicate ions may be able to induce angiogenesis. This was favored by the stimulation of the secretion of angiogenic growth factors such as vascular endothelial growth factors (VEGFs). Further in vitro and in vivo studies have discovered stimulating angiogenic effects of Si released from these materials, such as endothelial progenitor cell homing, migration, tubule formation, or vessel sprouting [40]. A possible pathway is the stimulation of hypoxic mechanisms by regulating the activity of hypoxia-related factors such as HIF-1α. Since angiogenesis and osteogenesis are closely coupled, these effects may also favor osteogenesis [40]. This dual function of Si in the stimulation of osteogenesis/angiogenesis is reflected in related effects of Si components used in bioceramics [54].

#### 2.2.5. Immune System

The influence of Si on the immune system is nearly unknown [24]. There have been only a few animal studies: After inducing inflammation in a collagen type II-injection model, rats with sufficient Si had lower lymphocytes and higher neutrophils [55]. An acute inflammatory induction with lipopolysaccharides (LPSes) led to an increase in Si concentrations in the liver and bone of rats with a Si shortage, while inflammatory markers were similar to animals with a normal Si substitution. Si deprivation did not affect acute-phase inflammatory markers or make changes induced by the injection of an endotoxin [56]. However, the uptake of Si by macrophages, as in the case of silicosis (see below), can lead to apoptosis [57]. Si is capable of producing reactive oxygen species that can trigger cell-signaling pathways to initiate cytokine production or apoptosis OC cells [58].

#### 2.2.6. Nervous System

Si may have a protective impact for dementias such as Alzheimer’s disease: The protective effect has been discussed as being related to aluminum binding of Si, forming aluminum silicates that can then be resorbed. Aluminum is thought to behave as a cofactor in the pathogenesis of Alzheimer’s dementia. Prospective cohort studies from France have investigated the interactions between aluminum and Si originating from drinking water in persons developing dementia or cognitive impairment over the years. High aluminum intake was correlated with a higher risk for developing dementia, while 10 mg of silica per day reduced the risk by 11% [59]. However, the negative effect of aluminum and the protective effect of silica was only observable for Alzheimer’s-induced dementia. In the Epidemiology of Osteoporosis study (EPIDOS), a correlation between the composition of drinking water and cognitive impairment was investigated in women older than 75 years. In the beginning, in women with normal cognitive functions, a higher Si input through water was noted in contrast to women with impaired cognitive functions. In a subgroup, regression analysis revealed a significant correlation between silicate input and Alzheimer’s risk at about 36%. Si supplementation came mostly from mineral waters [60].

### 2.3. Toxicity

According to the European Food Safety Authority (EFSA), there are no indications for toxic effects from Si even under high intake conditions. Higher dietary intakes “...are unlikely to cause adverse effects” [61]. In addition, in the context of the degradation of Si-containing bone substitute materials, no clear evidence for cytotoxicity, genotoxicity, or carcinogenicity has been reported. However, a transfection of Si ions from phagocytozing cells to the blood may be possible. Normally, the biocompatibility of Si-substituted bone ceramics, e.g., hydroxyapatites, is tested [62]. After the implantation of Si-containing bioglass in rabbit bone, Si was found to be excreted in the urine, but no higher amonts of the element were found in organs [63]. An evaluation of genotoxicity and cytotxicity in vitro of the same material revealed no inhibition of cellular proliferation and DNA damage. Histopathology after implantation in 65 rats showed granulation tissue after 7 days and fibrosis and multinucleated giant cell appearance after longer periods, but no necrosis [64]. However, chronic inhalation of crystalline Si is considered to be an occupational hazard and leads to silicosis, which may also be associated with the dysregulation of autoimmunity [58,65]. Si particles phagocytized by macrophages trigger inflammatory responses [1,66]. These toxic effects, especially in the airways, are also evident for Si nanoparticles, where they can induce inflammation [67]. For Si dioxide as a food additive, no indication for toxicity has been evaluated [68].

The known or proposed role of Si in the development, homeostasis, repair, and regeneraton of different tissues is the basis for using it as an element in biomaterials research. Due to its long-lasting and successful history in clinical applications, the first examples of using the beneficial functions of Si were bioglasses, recently reviewed by Drago et al. [69].

## 3. Mediated Calcium-Silicate Mineralization

### 3.1. Guided Calcium-Silicate Biomineralization

The development of new bone implant substitutes in the orthopedics field is focused on porous ceramic materials as one potential class of bone graft material. Maximizing structural and chemical properties are important objectives. The preferred bioceramic component is hydroxyapatite (HA), with a crystalline structure similar to the bone mineral. Hydroxyapatite has been studied extensively since the 1970s. It can bond to host bone and correspondingly to bioinert materials such as polymers, metals, and inert ceramic materials [70,71,72]. However, there are significant differences between synthetic HA and that formed by the body itself [73]. In addition to crystalline differences, artificial HA lacks many of the inorganic substances and trace elements incorporated into the form produced in vivo. The differences between endogenous HA and exogenous HA lead to altered resorption rates and prolonged repair periods [74,75,76]. In certain circumstances, this prolonged resorption may be advantageous, but in most situations it is preferable to have a graft material gradually replaced by host bone.

Other restorable bioceramics have been developed with differing resorption properties. For example, beta-tricalcium phosphate (β-TCP) and calcium sulphate (CS) have resorption rates of approximately six months and three weeks, respectively. Silicates have been demonstrated to be important for bone formation in dietary intake studies. Bone integration with silicate-substituted products has been shown to facilitate bone formation, providing solid evidence of its place as a new class of bone graft material [77,78,79,80,81].

Furthermore, the research area of biomineralization is closely related to approaches that use organic templates in order to create new functional materials with applications such as bone tissue. Materials that are obtained through synthetic routes inspired by nature, and that are therefore closely connected to biomineralization, are also called biomimetic materials. Most of these biomimetic syntheses are carried out under mild reaction conditions, e.g., almost neutral pH, room temperature, nontoxic reagents (templates), and aqueous media. While some templates are of natural origin or are directly obtained through natural resources [82], others are produced completely synthetically [83]. An example of semisynthetically produced templates are chitosan derivatives, since the raw materials are obtained from natural sources (e.g., the shells of crabs are refined or subsequently functionalized [84,85]). Synthetic or natural templates often show similarities regarding their functional groups. Among other things, very often carboxyl, phosphate, hydroxyl, or sulphate functionalities are commonly observed. In addition, nitrogen-containing groups such as amino (-NH2) or imidazole functionalities can also be found [86,87,88].

Organic templates influence several parameters of inorganic materials, e.g., the shape or morphology and phase composition of the concerned materials. In addition, templates that generate isolated inorganic or composite materials with geometric shapes such as tubes, needles, or cones will be discussed. Among them are structures of natural origin such as silicateins or chitosan, as well as synthetic compounds such as ionic liquids and polyamines. Since many reviews and publications are already known within this wide area of research, only the most recent publications and breakthroughs of major importance will be highlighted [3,46].

Additionally, a 3D relationship could be established between the areas of bone formation within animal tissue and the accumulation of silicon. Thus, a silicon increase has been seen around the osteoid and osteoid–bone interfaces, implying that silicate is essential for bone formation. Consequently, the effect of silicate, enzymatically catalyzed by silicatein, on the activity of osteoblasts has been investigated in depth. Indeed, the cell model used displayed an increased mineralization activity when cultivated on biosilica surfaces [89]. Some studies have revealed that the combination of ß-glycerophosphate and silica-based components increases the expression of these marker genes and is further supported by the increased deposition of hydroxyapatite crystallites on the surfaces of these cells [90,91].

The increased solubility compared to chitin may be explained by protonated amino groups, resulting in increased polarity and electrostatic repulsion. For that reason, chitin is deacetylated to chitosan, which leads to increased solubility and decreased crystallinity [92,93]. Chemical and physical modifications can enhance the solubility at higher pH levels. Due to reduced crystallinity and the presence of hydroxyl, amino, and acetamide functionalities, chitosan provides also an expendable backbone for further functionalization, depending on the intended application. Important derivatives are alkyl chitosan, N-(O)-acylchitosans, N-carboxyalkyl (aryl)-chitosans, thiolated chitosans, sugar-modified chitosans, and sulphated and phosphorylated chitosans [94]. Furthermore, reduced chain lengths up to oligomeric structures can also influence solubility. Therefore, chitosan provides a starting point for further research into the field of biocomposites, with potential practical applications such as drug delivery material [95,96], to better obtain mechanical stability [97] or to support tooth tissue regeneration or multilayer biomineralization, as reported by Leite et al. [98,99].

Chemical and physical modifications can enhance solubility at higher pH levels. Due to reduced crystallinity and the presence of hydroxyl, amino, and acetamide functionalities, chitosan provides also an expendable backbone for further functionalization, depending on the intended application. Important derivatives are alkyl chitosan, N-(O)-acylchitosans, N-carboxyalkyl (aryl)-chitosans, thiolated chitosans, sugar-modified chitosans, and sulphated and phosphorylated chitosans [85]. Furthermore, reduced chain lengths up to oligomeric structures and variations in the degree of deacetylation can also influence solubility. Therefore, chitosan provides a starting point for further research into the field of biocomposites, with potential practical applications.

Palmer et al. performed an in-lab biomineralization synthesis process with the addition of acidic collagen suspensions containing phosphate ions into a basic suspension under continuous stirring. Within a pH range from 4.5 to 6.5, they observed dispersed nanosized collagen fibrils start to assemble [100]. When collagen came into contact with Ca^2^+ ions, they soon linked to COO− groups exposed by C-terminal regions of tropocollagen fibrils, so that the formation of apatite crystals was initiated by the phenomena of heterogeneous nucleation. In the presence of additional foreign ions (Mg^2^+ and SiO_4_^−^) during in-lab biomineralization, the crystal disorder of the heterogeneously nucleated HA particles favored the incorporation and substitution of Ca^2+^ and PO_4_^3−^, respectively, in the apatite structure, thus forming a biomimetic HA strongly mimicking in composition the inorganic part of human bone [100,101,102,103]. Indeed, the interaction with the collagenous matrix at the nanoscale was mediated by ultrastructural, chemotactic, and physical constraints affecting crystal growth and organization of the mineral phase.

### 3.2. Detailed Structure Analysis Using X-Ray Techniques

The arrangement and orientation of a nanostructure play a fundamental role in determining the mechanical properties of inhomogeneous isotropic and anisotropic bone materials. Widely used microscopic methods (TEM, SEM) can provide only limited 3D information about biomaterials [104]. In contrast, small-angle X-ray scattering (SAXS) and wide-angle X-ray scattering (WAXS) methods are based on a microfocus X-ray and therefore offer raster scans for different rotation angles and provide local 3D orientation of the bone structure [105,106,107,108]. SAXS and WAXS are nondestructive methods for measuring the qualitative and quantitative composition of crystalline, semicrystalline, and amorphous components and for characterizing and quantifying layers and particle dimensions, as well as mechanical stress data. Crystallinity refers to the degree of structural order in a solid and has a significant influence on hardness, density, transparency, and diffusion. Even within materials that are completely crystalline, the degree of structural perfection can vary, reflecting size and elastic strain from the independent crystalline regions (grains or crystallites) of which these materials are composed.

Recently established microdiffraction sources and high-resolution 2D-XRD area detectors [109] enable the analysis of a tooth in situ without destruction. Moreover, it has been possible to apply this nondestructive technique in carious and normal selected areas [86]. Unfortunately, like all local techniques, 2D and 3D X-ray microdiffraction analysis has some drawbacks in the characterization of the human tooth. First, each natural tooth has its own individual structure, which does not repeat itself in other zones of the same tooth. Second, as is well known, the crystallites in human enamel are regularly arranged, so they produce an anisotropic X-ray intensity distribution that is designated as texture.

**Figure 1:**
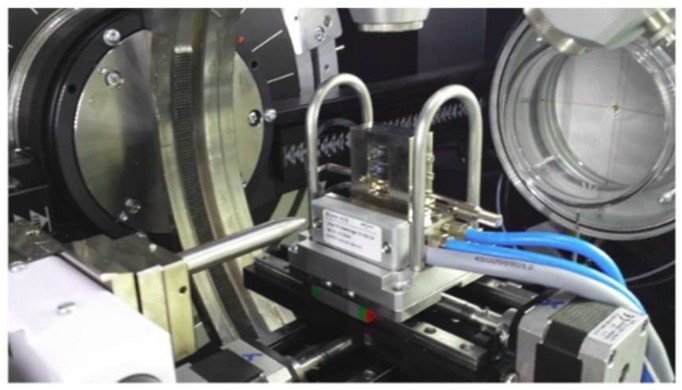
Setup of wide-angle X-ray scattering (WAXS) and small-angle X-ray scattering (SAXS).

WAXS is created from the interference of X-rays with electrons of atoms in lattices and nanoparticles. Scattering points with a characteristic size or spacing *d* reinforces scattered intensity in specific directions according to Bragg’s relationship nλ = 2*d* sinΘ, where λ is the X-ray wavelength, *d* is the difference between crystal lattice layers, and 2Θ is the angle between the incident and diffracted beam directions. The shape and broadening of XRD line profiles offer microstructural information containing the average size, size distribution, and shape of crystallites in the range of 5 to 100 nm; lattice defects; and the spatial arrangement of dislocations. The relation used for the calculation of crystallite size is the well-established Scherrer formula. With new high-brilliant X-ray sources and 2D detectors, wide-angle X-ray scattering (WAXS) and small-angle X-ray scattering (SAXS) can be combined into one instrument (Figure 1. Setup of wide-angle X-ray scattering (WAXS) and small-angle X-ray scattering (SAXS). SAXS is a powerful method for characterizing the micro- and nanostructure of a disordered heterogeneous biomaterial. This technique can be used to study wet samples and reactions in environmental conditions.

**Figure 2:**
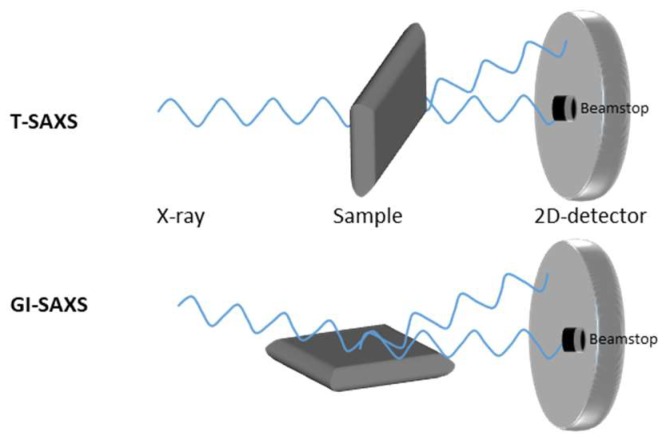
SAXS setup: Transmission (T)-SAXS and gracing incidence (GI)-SAXS measurement setup.

Small-angle scattering is possible in transmission mode (T-SAXS) and gracing incidence mode (GI-SAXS, Figure 2). A highly focused X-ray beam and an area detector are necessary for SAXS measurement. A T-SAXS setup is only possible with transparent samples. WAXS, SAXS, and microscopic measurements have shown that the characterized dimensions vary in the following ranges: Length (20–50 nm), width (15–30 nm), and thickness (1.5–4 nm) [110,111].

Bone is made of about 65 wt % mineral phase (nanosized crystals of apatite), 25 wt % organic phase (basically type-I collagen, noncollagenous proteins (NCPs), and minor organic molecules such as citrate), and 10 wt % water [112]. SAXS and WAXS can be used simultaneously to provide information on the unit crystals, crystal incorporations, crystal size, and collagen fibrils in bone [113]. SAXS especially has been established in the past few decades to investigate collagen fibril orientation in collagen-rich tissues.

In particular, the crystal structure of the mineral phase results in being characterized by an amorphous or very short-range order: In addition, the topotactic information provided by the collagenous matrix at the sites of heterogeneous nucleation induces preferential crystal growth of the HA-hexagonal crystals, with the *c* axis elongated along the long axis of collagen. As a consequence, the crystallographic *ab* plane of the newly formed HA phase is exposed perpendicularly to the long axis of the collagen fibers: This feature is supposed to promote the specific adsorption of proteins specifically involved in new bone formation [80].

SAXS/WAXS mapping also permits the measurement of position-resolved strontium content through slight changes in the apatite lattice constant. The measurement methods revealed in the investigations of Li et. al. have shown an advantage over fluorescence methods (also employed, for complementary information): Only strontium incorporated into the bone crystals was detected, as distinguished from strontium complexed or adsorbed into soft tissues [105]. According to these results, scanning SAXS/WAXS had the ability to probe the size and aspect ratio of nm-scale calcium phosphate mineral platelets, as well as to obtain a high-resolution measurement of the apatite lattice constant, which had a linear dependence on the strontium content incorporated into the structure. Moreover, by scanning thin slices of biopsied bone with a 15-μm beam spot, the team could distinguish regions of newly formed bone from areas of old bone formed before the treatment began.

With a 3D SAXS method using a microfocus X-ray beam from a synchrotron radiation source, a raster scan has been performed for sampling with different rotation angles. Additionally, a mathematical framework has been developed, validated, and employed to describe the relation between the SAXS data for the different rotation angles and the local 3D orientation and degree of orientation (DO) of the bone ultrastructure. The resulting local 3D orientation was visualized by a 3D orientation map using vector fields [114]. Using the SAXS/WAXS data, the study was able to conclude that strontium was incorporated only into newly formed bone crystals and showed no signs of affecting mineral ultrastructure. In the foregoing discussion, mention has not been made of the equally important effects of strain within materials, which also broadens diffraction peaks and which must be analyzed with care to properly interpret changes arising from strain or crystallite size. Using the Scherrer method, Salama et al. [115] detected that crystal size had a strong pH dependence on the crystallization of calcium phosphate nanoparticles through a coprecipitation method. The nature of the apatite layer formed in vitro and composed of different chitosan templates was characterized by XRD. Crystal formation and crystal size for different crystallization planes could be observed [116]. Mineralization kinetics of hydroxyl apatite during incubation times of 14 days were analyzed [98].

## 4. Si-Containing Scaffolds for Stem Cell-Based Bone Formation

### 4.1. Scaffold Manufacturing

Bone scaffolds have been receiving increased attention for use in stem cell-based tissue engineering approaches due to their influence in stem cell differentiation, vascularization, and the healing process. Besides providing support to the regenerating tissue, scaffolds simultaneously can be used to deliver bioactive molecules to accelerate the healing process. In a best-case scenario, scaffolds themselves show osteoinductive and/or osteoconductive effects. Thus, numerous efforts have been directed toward identifying the ideal scaffold material possessing all requisites for accelerated bone tissue regeneration. So far, materials exploited as scaffolds for orthopedic applications include polymers, metals, ceramics, and corresponding composites of organic and inorganic components (i.e., polymer/polymer, polymer/ceramic, polymer/metal, etc.) [117,118]. In the case of composite (also called hybrid) materials, bioinspired mineralization processes have been studied in detail to specify the importance of organic templates in inorganic structure formation [119,120]. Here, Karunya and Lechner have reported the preparation of novel biotemplated silica composites [121,122].

Today, stem cell-based approaches enable the development of individualized patient-specific solutions [123,124,125]. The first step in cell–scaffold interactions are adhesion processes related to intensive interactions on cell–biomaterial surfaces and interfaces. These interactions strongly depend on surface polarity (hydrophilic versus hydrophobic surfaces), surface roughness, and topography, in particular concerning any kind of alignment. The scaffold development starts with polymer synthesis using state-of-the-art polymerization techniques to achieve well-defined porous structures to enable cell ingrowth. Both the polymer bulk and surface have to be tailored to meet the needs of the natural environment. Scaffold surface polarity and topography have to be adapted to the cell shape in order to support cell adhesion, proliferation, and growth [126,127,128,129].

In general, there are various possibilities for introducing Si into a bone scaffold material: Often, aqueous Si solutions are used to study their dose-dependent enhancement of osteoblast proliferation and differentiation [130,131]. Another approach is the addition of orthosilicic acid to conditioned medium, resulting in a significant increase in human osteoblast formation. Thus, human osteoblast-like cells have been incubated with Si (3.6 mM) for 48 h, and a dose-dependent increase in proliferation and osteogenic differentiation mediated through upregulation of transforming growth factor beta was reported [132].

In Table 1, manufacturing methods of Si-containing scaffolds studied for use in hard tissue regeneration are summarized.

Mendes et al. prepared a variety of injectable bone β-TCP-monocalcium phosphate monohydrate (MCPM)-based cements, including mesoporous silica particles. Mesoporous silica particles provided better physicochemical properties compared to silica-free cements. For example, toxicity assays showed low Chinese hamster ovary (CHO)-K1 cell viability after treatment with more concentrated extracts (200 mg mL^−1^) [133].

Khan et al. comprehensively reviewed calcium phosphate-based bioactive ceramics containing silicon as a substituent or a dopant. In bioceramics, silicon significantly enhances different properties, including chemical surface structure, mechanical strength, bioactivity, and biocompatibility [134].

Patel et al. compared in vivo behavior of pristine and Si-substituted hydroxyapatite granules and demonstrated that the bioactivity of hydroxyapatite was considerably improved by the inclusion of silicate ions into its lattice. In addition, osteoblast cell activity was significantly enhanced by the substitution of phosphate (PO_4_^3−^) ions with silicate (SiO_4_^4−^) ions in hydroxyapatite [135].

So far, the influence of Si on the osteogenic differentiation of mesenchymal stem cells is not yet fully understood. Pietak and colleagues have reported bone healing studies involving the application of Si-substituted calcium phosphates (i.e., Si/HA, Si/TCP), confirming a significant influence on biological performance. However, no direct correlation could be found between Si release and the improved biological performance of Si-substituted calcium phosphates (CaPs) [2]. Ten years later, Wang and colleagues reported various options for Si incorporation and influence: Most importantly, silicon counterions and/or ligands did alter Si release into the surrounding tissue. In addition, the specific constitution of the Si-containing derivatives changed the dissolution rate of Si composites, their particle and pore size, and their size distribution, and thereby influenced the entire composite surface and topography. Thus, the authors concluded that the bioactivity of Si was attributed to the leaching and accumulation of silicon ions when exposed to body fluids upon implantation and the subsequent formation of a hydroxyapatite coating on the surface [139].

**Figure 3:**
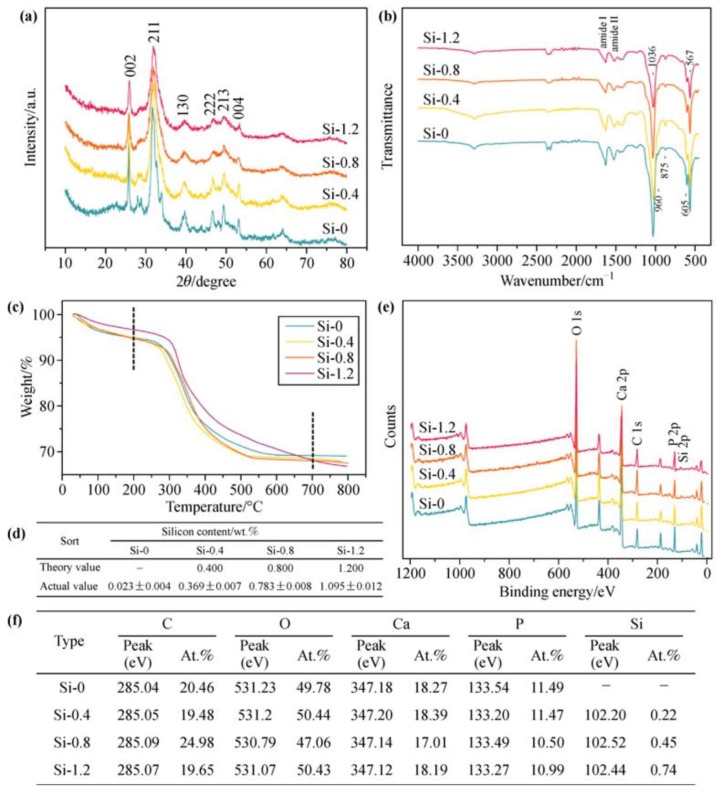
Characterization of materials. (**a**) XRD analysis showing that both Si-free and Si-doped HA groups exhibited the main diffraction peaks of HA in comparison to the HA standard card (JCPDS-PDF 09-0432). (**b**) FTIR analysis indicating that each group of template-induced materials displayed the typical phosphate group and main functional groups of amides I and II due to the presence of organic protein templates. (**c**) Thermogravimetric (TGA) analysis confirming that the template-induced materials were organic–inorganic composites consisting of 20%–30% organics and 70%–80% inorganics. (**d**) Si-molybdenum blue spectrophotometry confirming that the actual value of silicon content was quite close to the theory value. (**e**,**f**) X-ray photoelectron spectroscopy (XPS) analysis showing the typical peaks of Ca and P in a Si–O group and the typical peaks of Si in Si-doped groups. The mole ratio of n(Ca)/n(P) or n(Ca)/[n(P) + n(Si)] of the sample was close to the theoretical value of HA (n(Ca)/n(P) = 1.67). Reprinted from [140], with permission from Springer Nature, 2017.

Yang et al. have reported a series of biomimetic Si-doped bone composites fabricated via template-mediated synthesis. Due to their biocompatibility, the adhesion and proliferation of mesenchymal stem cells was observed, resulting in induced osteoblast formation. The fate of Si-doped powder materials in vivo was observed by introduction of polylactic-*co*-glycolic acid (PLGA) to fabricate porous and lamellar scaffolds through a freezing cast technique. Si-0 or Si-0.8 powders of 100 μm in diameter were added to the PLGA solution. During the freezing process, lamellar ice crystals grew preferentially from the metal base to the top, generating a gradient in their thickness. Three groups of PLGA, PLGA/Si-0, and PLGA/Si-0.8 were prepared for animal tests in vivo. According to the authors, this study provided a feasible protocol to combine element doping with a template-mediated strategy to generate novel hybrid composites. Figure 3 gives an overview on scaffold analysis using X-ray diffraction (XRD), Fourier-transform infrared (FTIR) spectroscopy, thermogravimetric analysis (TGA), and X-ray photoelectron spectroscopy (XPS) methods, thereby proving Si’s influence on scaffold quality [140].

In 2015, Ferro De Godoy and colleagues verified the osteostimulative potential of a new formulation of Si-containing calcium phosphates with enhanced porosity. This osteostimulating effect was attributed to the materials’ microstructures and chemistry [141]. In 2016, Wang and colleagues studied the dual role and dose-dependent effects of silicates in the crystallization of hydroxyapatite in simulated body fluids. They found that at lower silicate concentrations (0.05–0.5 mM), the nucleation of hydroxy apatite was promoted, and the lower the amount of silicate, the faster the HA nucleation. In contrast, at higher silicate concentrations (3–8 mM), the nucleation of HA was inhibited [142]. In 2017, Sadeghzade and colleagues studied for the first time the potential of a space holder method as a new technique to fabricate diopside/forsterite scaffolds. Diopsides are monoclinic pyroxene minerals (MgCaSi_2_O_6_) forming complete solid solutions with hedenbergite (FeCaSi_2_O_6_), while forsterite is composed of magnesium, oxygen, and silicon (Mg_2_SiO_4_). Those space holders are required, since one of the main challenges of using scaffolds in bone defects is the mechanical strength mismatch between the implant and surrounding host tissue, which causes stress shielding or failure of the implant during the course of treatment. During the sintering process, NaCl, as a spacer agent, gradually evaporated from the system and produced desirable pore sizes in the scaffolds. The results showed that adding 10 wt % of diopside to forsterite could enormously improve the bioactivity, biodegradability, and mechanical properties of the composite scaffolds. The sizes of the crystals and pores of the obtained scaffolds were measured to be in the range of 70–100 nm and 100–250 μm, respectively. Composite scaffolds containing 10 wt % of diopside showed a similar compressive strength and Young’s modulus (4.36 ± 0.3 and 308.15 ± 7 MPa) to bone. Moreover, the effects of adding various diopside contents on the mechanical and physical aspects, size of crystals, bioactivity, and biodegradability of forsterite ceramics were investigated. Diopside acts as a sintering aid and decreases the required sintering temperature and subsequently reduces grain growth that occurs during the sintering process. In addition, it has a higher bioactivity compared to forsterite, and therefore it can provide a biological fixation in a shorter period of time, which prevents the movement and failure of the implant [143]. Belton and colleagues have studied biosilicification processes in detail and have described some basic dependencies regarding silica precursors and technologies, including the aqueous chemistry of silica, from uncondensed monomers through colloidal particles and 3D structures, i.e., Si alkoxides such as tetraethoxysilane and silicatein [144].

### 4.2. Nanostructured Si-Containing Scaffolds

Additive manufacturing methods for bone scaffold materials, recently reviewed by Schipper et al., include the following main techniques [145]:

- Rapid prototyping (RP) methods such as selective laser sintering (SLS), selective laser ablation (SLA), and fused deposition modeling (FDM) [146];
- Electrospinning methods to form fibers of various diameters [147];
- Chemical and physical vapor deposition (CVD, PVD) for surface functionalization and modification, i.e., hydrophobic versus hydrophilic surfaces [148];
- 3D printing methods resulting in tailor-made layered (a), cubic (b), and spherical (c) structures [149,150];
- Self-assembly methods, e.g., the Langmuir–Blodgett technique for monolayer formation, including (d) the spreading of polymer solution, (e) compression to a single monolayer, and (f) film transformation onto substrates; and electrospinning rigid (g) and flexible (h) polymers [151].

The discovery of fullerenes and carbon nanotubes induced a tremendous development in novel nanomaterials and investigations into their use in many different applications [152]. Nanostructured biomaterials including nanoparticles, nanofibers, nanosurfaces, nanocomposites, and nanosphere-immobilized biomaterials have gained increasing interest in regenerative medicine, since these materials often mimic the extracellular matrix (ECM). Thus, nanomaterials have been intensively studied in the last decade for utilization in tissue engineering and scaffold fabrication, particularly for bone regeneration. Materials that have been designed on the nanoscale that have been used for bone regeneration mainly include the following:

- Nanospheres and nanoparticles [153,154,155,156,157];
- Nanotubes, in particular carbon nanotubes [148];
- Nanodendrimers based on carboxymethylchitosan/poly(amido amine) [158].

Nanoscaled silicon-containing materials have been studied by various research groups [159,160]. Shadjou has summarized recent progress in bone tissue engineering using silica-based mesoporous nanomaterials possessing pore sizes in the range of 2–50 nm and surface reactive functionalities [159].

**Figure 4:**
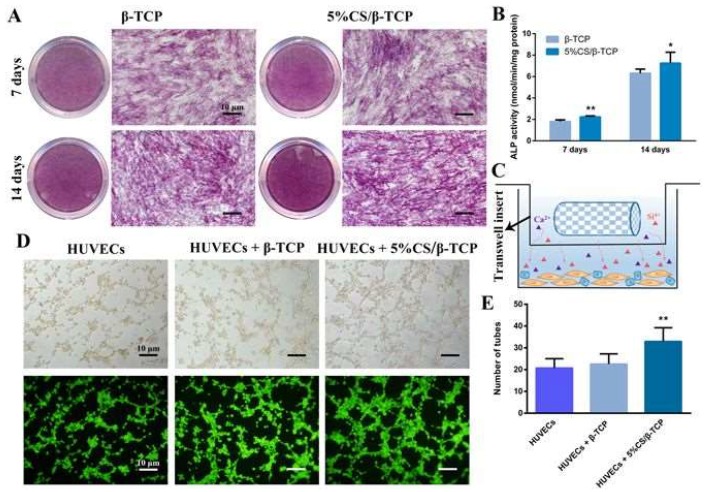
Five percent calcium silicate (CS)/β-TCP scaffolds stimulated human bone marrow stromal cell (hBMSC) osteogenesis and human umbilical cord vein endothelial cell (HUVEC) angiogenesis in vitro. (**A**) Alkaline phosphatase (ALP) staining in hBMSCs cultured for 7 and 14 days with β-TCP scaffolds or 5% CS/β-TCP scaffolds in transwell inserts. (**B**) 5% CS/β-TCP scaffolds stimulated ALP activity at 7 days and 14 days in comparison to pure β-TCP. (**C**) Schematic representation of transwell experiments. Scaffolds in transwell inserts (upper chamber), cells in the lower chamber. (**D**) Tube formation by HUVECs, as observed by light microscopy (top) and calcein acetoxymethyl (AM) staining (bottom), after 4 h on Matrigel with β-TCP or 5% CS/β-TCP scaffolds in transwell inserts; (**E**) 5% CS/β-TCP scaffolds enhanced tube formation in comparison to β-TCP scaffolds. * *p* < 0.05; ** *p* < 0.01. Scale bar represents 10 μm. Reprinted from [161], published under a Creative Commons Attribution 4.0 International License, 2017.

Synthetic bone scaffolds have potential applications in repairing large bone defects: However, inefficient vascularization after implantation remains the major issue of graft failure. Porous β-tricalcium phosphate (β-TCP) scaffolds with calcium silicate (CS) were 3D-printed and pre-seeded with cocultured human umbilical cord vein endothelial cells (HUVECs) and human bone marrow stromal cells (hBMSCs) to construct tissue engineering scaffolds with accelerated vascularization and better bone formation. The results showed that in vitro β-TCP scaffolds doped with 5% CS (5% CS/β-TCP) were biocompatible and stimulated angiogenesis and osteogenesis. The results also showed that 5% CS/β-TCP scaffolds not only stimulated cocultured cell angiogenesis on Matrigel, but also stimulated cocultured cells to form microcapillary-like structures on scaffolds and promoted the migration of BMSCs by stimulating cocultured cells to secrete platelet-derived growth factor (PDGF)-BB and chemokine 12 CXCL12 into the surrounding environment. Moreover, 5% CS/β-TCP scaffolds enhanced vascularization and osteoinduction in comparison to β-TCP and synergized with cocultured cells to further increase early vessel formation, which was accompanied by earlier and better ectopic bone formation when implanted subcutaneously in nude mice. Thus, the findings suggest that porous 5% CS/β-TCP scaffolds seeded with cocultured cells provide a new strategy for accelerating tissue engineering scaffold vascularization and osteogenesis and show potential as a treatment for large bone defects (Figure 4) [161].

A wide range of techniques are being used to probe the complex structure of scaffold materials at different levels, from a micrometer- to a nanometer-length scale and down to their molecular structures [162,163]. In addition to analytical methods for structure analysis discussed before (SAXS, WAXS, FTIR, and Raman) nuclear magnetic resonance (NMR) spectroscopy is also used for characterizing silica species in solution and in the solid state: Silicon species are identified as 29Si nuclei. At the molecular level, solid-state ^29^Si NMR allows for the observation of connectivity and degrees of condensation in solid siliceous materials by comparing the intensities of various signals. In their review, Zhou and colleagues provide a brief history summarizing first attempts and recent results, including contradictory reports using silicates in the development of biomaterials for tissue engineering, particularly silicate-based glasses and ceramics for bone grafts [131].

Today, analytical platforms are developed to holistically prove the authenticity of natural products isolated from animal and/or plant raw materials. Thus, modern analytical methods are currently combined with multivariate data processing. Chemometric modeling of 2D NMR spectra (i.e., diffusion-ordered spectroscopy (DOSY), heteronuclear single-quantum correlation spectroscopy (HSQC), heteronuclear multiple-bond correlation spectroscopy (HMBC)) is reported using principal component analysis (PCA), independent component analysis (ICA), multivariate regression (PLS), and various discriminant analysis methods (i.e., linear discrimination analysis (LDA), partial least squares regression discrimination analysis (PLS-DA)). Quantitative characteristics (molecular weight, the content of active ingredients and impurities, pharmacological activity, etc.) and qualitative properties (plant origin, genotype, phenotype, manufacturer) can be determined based on spectrometric and chromatographic profiles, which can be shown for heparins of different origins using 2D NMR and size exclusion chromatography (SEC) data [164]. This approach is universal and can be applied to other methods and products as well. Empirical techniques have evolved into statistical approaches (i.e., FTIR, NMR).

### 4.3. Commercial Products and Patents

The relevance of silicate and related compounds is visible in a number of commercial products and filed patents reporting the effect of silicon in bone regeneration (Table 2).

The patent US 20170088431 A1 [168] claims a method for synthesizing calcium-silicate-based porous particles with controlled morphology and pore size achieved through a refined solution-based synthesis, allowing loading of a variety of sealants. These particles, upon external stimuli, release the loaded sealant into the surrounding material. The materials claimed are calcium-silicate-based porous particles comprising a calcium-silicate structure with pores located on the exterior surface, and an interior network of pores formed throughout the composite structure. Particle size varies between 50 nm and 2 µm in average diameter. The calcium-to-silicon atomic ratio varies between 0.05 and 2.0, with pores having a diameter size of 1–50 nanometers in average diameter.

Mesoporous calcium silicate compositions and methods for the synthesis of mesoporous calcium silicate for the controlled release of bioactive agents are disclosed in US 9539359 B2 [169]. In one embodiment, mesoporous calcium silicate was synthesized by acid modification of wollastonite particles using hydrochloric acid. A hydrated silica gel layer having abundant Si–OH functional groups can be formed on the surface of wollastonite after acid modification. The Bruhauer–Emmett–Teller (BET) surface area increased significantly due to acid modification and, in one arrangement, reached over 350 m^2^/g. Acid-modified mesoporous calcium silicate compositions showed a higher ability to adsorb protein compared to unmodified particles and demonstrated controlled release kinetics of these proteins.

In vivo 3D printing of regenerative bone healing scaffolds for rapid fracture healing is disclosed in the patent US 20170143831 A1 [170]: 3D tissue repair and regeneration through precise and specific formation of biodegradable tissue scaffolds in a tissue site using bio-inks are also provided. Specific methylacrylated gelatin hydrogels and methacrylated chitosan preparations formulated with sucrose, a silicate-containing component (such as laponite) and/or a cross-linking agent (such as a photo-initiator or chemical initiator), as well as powdered preparations of these, are also disclosed. Kits containing these preparations are provided for point-of-care tissue repair in vivo. Superior, more complete (up to 99.85% tissue regeneration within four weeks applied in situ), and rapid in situ tissue repair and bone formation are also demonstrated.

US 20160106885 A1 [171] claims a synthetic composite material for tissue repair comprising two layers: A first layer comprising an organic material and having side walls and an external surface, and a second porous layer comprising an inorganic material and having side walls, wherein the first layer is in direct contact with the second layer and wherein the side walls of the first layer and the side walls of the second layer are coated with a third layer comprising the organic material (i.e., hydroxyapatite, calcium sulphate, calcium silicate, calcium phosphate, magnesium silicate, and metal, preferably magnesium or titanium, Poly(D,L-lactide-*co*-glycolide), or a combination thereof).

US 9327976B2 [172] claims a hydroxyapatite multisubstituted with physiologically compatible ion species and its biohybrid composite with a natural and/or synthetic polymer, which are useful in the preparation of a biomimetic bone substitute for treating bone tissue defects. Furthermore, the present invention relates to a method for its preparation and use. Among the most important ion substituents are silicate and carbonate anions, which partially substitute phosphate ions (so-called “site B” of HA): Mg and Sr partially substitute calcium ions.

### 4.4. Si-Containing Drug Release Materials in Bone Regeneration

Within the last five years, calcium phosphate cements and HA-derived hybrid materials have gained increasing interest, since they have been shown to combine scaffold function with the additional ability of sustained release, which has been studied in detail for a number of different drugs, including low molecular weight (i.e.*,* antibiotics, anticancer drugs) and high molecular weight compounds (proteins, growth factors) and ions (i.e.*,* Ca, Sr, Si, Zn, Mg) [173]. Due to their osteoconductivity and injectability, they are already used as bone grafts. Moreover, their low-temperature setting reaction and intrinsic porosity allow drug incorporation and release. The osteogenesis of MSCs is known to be influenced by a broad number of osteoinductive and osteoconductive compounds, such as growth factors. Recently, it could be shown that stem cell differentiation toward bone is guided by various purinergic receptors (P2X and P2Y) [174,175,176,177]. Thus, the controlled release of corresponding P2 ligands (agonists, antagonists) encapsulated and kinetically controlled and released during MSC differentiation could be used to tailor MSC differentiation into cardiovascular and bone tissue [178,179,180,181]. The first hybrid materials based on HA and polysaccharides (agarose derivatives) have been prepared for use as appropriate scaffolding for MSC differentiation and guided ligand release [182].

In addition to conventional raw materials used for drug encapsulation, novel biobased raw materials are available from biorefineries using lignocellulose feedstocks [183,184,185]. Here, silicone-containing biopolymers at the micro- and nanoscale are promising candidates to be studied as materials for drug encapsulation and sustained release [186,187].

Very recently, Xing et al. reported an approach of combining silicon (Si) and strontium (Sr) ions released from bioceramic hydrogels to study their effect on the proliferation and osteogenic differentiation of human bone marrow-derived mesenchymal stem cells [138]. Thus, the obtained results showed that Si and Sr ions could synergistically stimulate cell proliferation and stimulate osteogenic differentiation. In addition, the authors could show that blood vessel formation and angiogenesis were enhanced.

A comprehensive review was reported by Trofimov et al. providing detailed information on the synthesis of inorganic porous silica-based particles. A special focus was placed on loading capacity and controllable drug release affected by silicon derivatives. Detailed structure–property relationships were presented, including internal biological stimuli (e.g., pH, redox, enzymes) and external noninvasive stimuli (e.g., light, magnetic field, and ultrasound) [156,188].

## 5. Future Aspects

Silica-based materials have been recognized regarding their bio-inertness and biocompatibility. In the last decade, various studies have confirmed positive effects on bone formation. Thus, elemental silicon and Si-containing compounds will be studied in greater detail regarding their osteoconductive and/or osteoinductive capacity. Here, further analytical studies are required in order to explain the correlation between the 3D structure and/or polarity of the silicon derivatives and their influence on osteogenesis.

Furthermore, silica-precipitating molecules can result in a variety of nanostructured formations, depending on the reaction conditions and additives. Here, detailed analytical studies using modern techniques such as X-ray and neutron scattering are required regarding the elucidation of the particular 3D structures and surfaces of those nanostructures. Based on structural information, specific structure–property relationships can be developed in order to understand and explain the detailed mechanisms of the observed effects. In addition, biotemplated self-assembled Si micro- and nanocomposites have been shown to be potential replacements for chemically synthesized silicates and may become appropriate materials for scaffold development in hard tissue engineering.

## Figures and Tables

**Table 1:** Manufacturing methods of Si-containing bone scaffolds. TCP: Tricalcium phosphate; MCPM: Monocalcium phosphate monohydrate; HA: Hydroxy apatite.

Scaffolds	Composition	Manufacturing	Reference
**Si-containing calcium phosphate cements**	Injectable β-TCP-based cements with mesoporous Si particles (monocalcium phosphate monohydrate, MCPM)	β-TCP/MCPM/Si with 5% silica and a solution of polyethylene glycol 400 in deionized water (1:1, *v*/*v*) as a liquid component in the preparation of the cements	Mendes et al., 2017 [133]
**Si-containing ceramics**	Si-substituted calcium phosphate-based bioceramics HA/0.8 wt % Si-substituted HA granules	Reviewing various preparation methods and mechanism of bone bonding to calcium phosphate Si-containing bioceramics Prepared by aqueous precipitation and processed into granules of 0.5–1.0 mm in diameter, sintered at 1200 °C	Khan et al., 2014 [134] Patel et al., 2002 [135]
**Si-containing bioglass**	Bioglass 45S5^®^ (46.1 mol % SiO_2_, 24.4 mol % Na_2_O, 26.9 mol % CaO, 2.6 mol % P_2_O_5_), NovaBone Products LLC, US S53P4^®^ (53.8 mol % SiO_2_, 22.7 mol % Na_2_O, 21.8 mol % CaO, 1.7 mol % P_2_O_5_), BonAlive Biomaterials, Finland	Modern sol-gel techniques to introduce pores of various sizes	Gaisser et al., 2013 [136]
	Synthetic silicate-based ceramics, originally SiO_2_, Na_2_O, CaO, P_2_O_5_		Pallan et al., 2016 [137]
	Silicate-based ceramics including strontium ions		Xing et al., 2018 [138]

**Table 2:** Commercial scaffolds, their composition, and application area.

Product	Origin	Application	Description	Reference
Actifuse^TM^	artificial	oral reconstruction, bone augmentation	76% nanocrystalline Ca phosphate plus 24% SiO_2_; 80% porosity; osteoinductive	case study: Jenis et al., 2010 [165]
BONITmatrix^®^	artificial	oral reconstruction, bone augmentation	nanocrystalline HA (60%), ß-TCP (40%); sol-gel mixture (87:13) in SiO_2_ matrix, interconnective pores, osteoconductive	Gredes et al., 2012 [166]
Nanos^®^	artificial	extraction defect restoration, oral reconstruction, bone augmentation	nanocrystalline Ca phosphate in SiO_2_ matrix (no sintering); osteoconductive	Brinkmann et al., 2017 [167]

## Abbreviations

ALP	alkaline phosphatase
AM	acetoxymethyl
BET	Bruhauer–Emmett–Teller
BMD	bone mineral density
BMP-2	bone morphogenetic protein-2
CaP	Calcium phosphate
CHO	Chinese hamster ovary
DOSY	diffusion-ordered spectroscopy
ECM	extracellular matrix
EFSA	European Food Safety Authority
EPIDOS	epidemiology of osteoporosis
ERK	extracellular signal–regulated kinases
FDA	Food and Drug Administration
FDM	fused deposition modeling
FTIR	Fourier-transform infrared spectroscopy
GI-SAXS	gracing incidence small angle X-ray scattering
HA	hydroxy apatite
HMBC	heteronuclear multiple-bond correlation spectroscopy
HSQC	heteronuclear single-quantum correlation spectroscopy
HUVEC	human umbilical cord vein endothelial cells
LB	Langmuir–Blodgett
LDA	linear discrimination analysis
LPS	lipopolysaccharides
MCPM	monocalcium phosphate monohydrate
MSC	mesenchymal stem cells
NCPs	noncollagenous proteins
NMR	nuclear magnetic resonance
OPG	osteoprotegerin
PDGF	platelet-derived growth factor
PINP	procollagen Type 1 N-terminal propeptide
PLS-DA	partial least squares regression discrimination analysis
RANK	receptor activator of nuclear factor κB
RANKL	receptor activator of nuclear factor κB ligand
RP	rapid prototyping
SLS	selective laser sintering
SLA	selective laser ablation
SAXS	small-angle X-ray scattering
SEC	size exclusion chromatography
Si	silicon
SiO_2_	silicon oxide
ß-TCP	beta-tricalcium phosphate
TGA	thermogravimetric analysis
T-SAXS	transition mode SAXS
VEGF	vascular endothelial growth factors
WAXS	wide-angle X-ray scattering
XPS	X-ray photon spectroscopy
XRD	X-ray diffraction
